# Supplementary material for: SARS-CoV-2 from COVID-19 Patients in the Republic of Moldova: Whole-Genome Sequencing Results
Source: Viruses. 2022 Oct 21;14(10):2310. doi: 10.3390/v14102310 (PMC9610950; doi:10.3390/v14102310)
Supplement: Supplementary file 1 [file viruses-14-02310-s001.zip › viruses-1894124-supplementary.pdf]

Supplementary Table S1: Overview of detected SNSs (single nucleotide substitutions) in Delta whole-genome SARS-Cov-2 isolates from the Republic of Moldova.

Genomic position in the reference genome	Nucleotide in the reference genome hCoV-19/Wuhan/WIV04/2019|EPI_ISL_402124	Nucleotide in the samples from Republic of Moldova	Genomic region	Number of occurrences of a mutation	Amino acid substitution
**186**	C	T	ORF1a	1	----
**193**	C	T	ORF1a	1	----
**203**	C	T	ORF1a	1	----
**210**	G	T	ORF1a	45	----
**213**	G	T	ORF1a	1	----
**241**	C	T	ORF1a	47	----
**361**	A	G	ORF1a	1	----
**521**	G	A	ORF1a	1	86 V>I
**526**	G	T	ORF1a	3	87 E>D
**687**	A	G	ORF1a	1	141 K>R
**745**	C	T	ORF1a	1	----
**1048**	G	T	ORF1a	25	261 K>N
**1191**	C	T	ORF1a	1	309 P>L
**1267**	C	T	ORF1a	1	----
**1274**	G	T	ORF1a	1	337 V>F
**1514**	C	T	ORF1a	1	417 H>Y
**1625**	C	T	ORF1a	1	454 L>F
**1667**	A	G	ORF1a	1	468 K>E
**1758**	C	T	ORF1a	1	498 A>V
**1820**	G	A	ORF1a	2	519 G>S
**1824**	C	T	ORF1a	1	520 A>V
**1877**	T	C	ORF1a	1	538 S>P
**1912**	C	T	ORF1a	1	----
**2144**	G	T	ORF1a	1	627 V>F
**2494**	G	T	ORF1a	1	743 E>D
**2518**	G	T	ORF1a	3	----
**2539**	G	A	ORF1a	1	----
**2623**	C	T	ORF1a	1	----
**2746**	T	C	ORF1a	1	----

**2908**	A	G	ORF1a	2	----
**3037**	C	T	ORF1a	48	----
**3066**	G	A	ORF1a	1	934 G>D
**3085**	G	T	ORF1a	1	940 E>D
**3092**	C	T	ORF1a	1	943 P>S
**3329**	A	C	ORF1a	1	1022 T>P
**3685**	G	A	ORF1a	4	----
**3807**	A	G	ORF1a	1	1181 N>S
**3871**	G	T	ORF1a	1	1202 K>N
**3923**	C	A	ORF1a	1	1220 P>T
**4084**	C	T	ORF1a	1	----
**4181**	G	T	ORF1a	46	1306 A>S
**4237**	T	C	ORF1a	5	----
**4300**	G	T	ORF1a	1	----
**4438**	C	T	ORF1a	1	----
**4777**	C	T	ORF1a	1	----
**4794**	C	T	ORF1a	1	1510 S>F
**4916**	A	G	ORF1a	1	1551 I>V
**5045**	T	G	ORF1a	1	1594 F>V
**5184**	C	T	ORF1a	1	1640 P>L
**5203**	C	T	ORF1a	1	----
**5206**	G	T	ORF1a	1	1647 M>I
**5437**	G	T	ORF1a	3	1724 E>D
**5467**	C	T	ORF1a	3	----
**5584**	A	G	ORF1a	1	----
**5629**	G	T	ORF1a	1	----
**5668**	G	T	ORF1a	1	1801 E>D
**5886**	C	T	ORF1a	1	1874 T>I
**6056**	G	A	ORF1a	1	1931 V>I
**6094**	G	T	ORF1a	1	1943 Q>H
**6401**	C	T	ORF1a	1	----
**6402**	C	T	ORF1a	46	2046 P>L
**6445**	C	T	ORF1a	1	----
**6730**	C	T	ORF1a	1	----
**6941**	C	T	ORF1a	1	----
**6968**	C	T	ORF1a	1	----
**7124**	C	T	ORF1a	46	2287 P>S
**7190**	A	G	ORF1a	1	2309 I>V

**7420**	C	T	ORF1a	2	----
**7564**	C	T	ORF1a	2	----
**7768**	C	T	ORF1a	3	----
**7851**	C	T	ORF1a	11	2529 A>V
**7936**	G	T	ORF1a	1	----
**7976**	T	C	ORF1a	1	----
**7976**	T	C	ORF1a	4	----
**8240**	C	T	ORF1a	1	2659 H>Y
**8247**	C	T	ORF1a	1	2661 S>F
**8929**	T	C	ORF1a	1	----
**8982**	C	T	ORF1a	1	----
**8986**	C	T	ORF1a	46	----
**9053**	G	T	ORF1a	46	2930 V>L
**9532**	C	T	ORF1a	1	----
**9712**	C	T	ORF1a	1	----
**9891**	C	T	ORF1a	2	3209 A>V
**10029**	C	T	ORF1a	44	3255 T>I
**10078**	C	T	ORF1a	44	----
**10761**	A	G	ORF1a	1	3449 K>R
**11201**	A	G	ORF1a	46	3646 T>A
**11270**	A	G	ORF1a	1	3669 M>V
**11299**	G	T	ORF1a	1	3678 K>N
**11300**	C	T	ORF1a	1	----
**11332**	A	G	ORF1a	46	----
**11418**	T	C	ORF1a	2	3718 V>A
**11514**	C	T	ORF1a	1	3750 T>I
**12118**	T	G	ORF1a	1	----
**12514**	G	T	ORF1a	1	----
**12526**	A	T	ORF1a	1	----
**12946**	T	C	ORF1a	1	----
**13019**	C	T	ORF1a	1	----
**13078**	T	C	ORF1a	1	----
**13136**	A	G	ORF1a	1	4291 I>V
**13210**	T	C	ORF1a	1	----
**13458**	C	T	ORF1a	1	4398 S>L
**13712**	A	G	ORF1b	1	82 K>R
**13981**	C	T	ORF1b	1	172 R>C
**14187**	G	A	ORF1b	1	----

**14318**	C	T	ORF1b	1	284 T>I
**14408**	C	G	ORF1b	4	314 P>R
**14408**	C	T	ORF1b	44	314 P>L
**14925**	C	T	ORF1b	1	----
**15380**	G	T	ORF1b	1	638 S>I
**15451**	G	A	ORF1b	48	662 G>S
**15543**	G	T	ORF1b	2	----
**15714**	C	T	ORF1b	1	----
**15735**	T	C	ORF1b	1	----
**15763**	C	T	ORF1b	5	----
**15848**	C	T	ORF1b	1	794 T>I
**15908**	G	T	ORF1b	1	814 G>V
**15952**	C	A	ORF1b	2	829 L>I
**15958**	G	T	ORF1b	2	831 A>S
**16111**	C	T	ORF1b	1	----
**16248**	T	A	ORF1b	1	----
**16329**	C	T	ORF1b	1	----
**16393**	C	T	ORF1b	1	976 P>S
**16466**	C	T	ORF1b	48	1000 P>L
**16538**	A	G	ORF1b	1	1024 D>G
**16733**	C	T	ORF1b	1	1089 S>L
**17019**	G	A	ORF1b	1	----
**17025**	T	C	ORF1b	1	----
**17040**	T	C	ORF1b	11	----
**17156**	C	T	ORF1b	1	1230 T>I
**17371**	G	T	ORF1b	1	1302 A>S
**17999**	C	T	ORF1b	1	1511 T>I
**18167**	C	T	ORF1b	1	1567 P>L
**18176**	C	T	ORF1b	1	1570 P>L
**18526**	C	T	ORF1b	1	1687 R>C
**18693**	C	T	ORF1b	1	----
**18695**	C	T	ORF1b	1	1743 T>I
**18713**	C	T	ORF1b	1	1749 A>V
**18744**	C	T	ORF1b	3	----
**18816**	G	A	ORF1b	9	----
**18828**	C	T	ORF1b	1	----
**18905**	G	A	ORF1b	1	1813 R>H
**19107**	T	C	ORF1b	1	----

**19160**	C	T	ORF1b	1	1898 S>F
**19219**	G	T	ORF1b	1	----
**19220**	C	T	ORF1b	46	1918 A>V
**19273**	C	T	ORF1b	1	1936 P>S
**19512**	C	T	ORF1b	1	----
**19524**	C	T	ORF1b	3	----
**19641**	T	C	ORF1b	1	----
**19872**	G	T	ORF1b	2	----
**19875**	C	T	ORF1b	1	----
**19983**	C	T	ORF1b	1	----
**20055**	A	G	ORF1b	1	----
**20057**	G	T	ORF1b	1	----
**20060**	G	T	ORF1b	7	2198 S>I
**20133**	C	T	ORF1b	4	----
**20247**	G	T	ORF1b	2	2260 M>I
**20262**	A	G	ORF1b	1	----
**20325**	C	T	ORF1b	1	----
**20438**	G	T	ORF1b	1	2324 S>I
**20523**	T	C	ORF1b	1	----
**20709**	G	T	ORF1b	1	2414 M>I
**20880**	T	A	ORF1b	1	----
**20937**	G	T	ORF1b	5	----
**21034**	C	T	ORF1b	1	2523 L>F
**21137**	A	G	ORF1b	2	2557 K>R
**21334**	G	T	ORF1b	5	2623 A>S
**21409**	C	T	ORF1b	1	2648 P>S
**21618**	C	G	S	48	19 T>R
**21647**	A	G	S	1	29 T>A
**21721**	C	T	S	1	----
**21846**	C	T	S	14	95 T>I
**21859**	C	T	S	1	----
**21976**	T	C	S	1	----
**21987**	G	A	S	48	142 G>D
**21995**	T	C	S	7	145 Y>H
**22017**	G	T	S	1	----
**22206**	A	G	S	1	215 D>G
**22225**	G	T	S	1	----
**22227**	C	T	S	8	222 A>V

**22311**	C	T	S	1	250 T>I
**22314**	C	T	S	3	251 P>L
**22458**	C	T	S	1	299 T>I
**22792**	C	T	S	1	----
**22917**	T	G	S	48	452 L>R
**22993**	C	T	S	1	
**22995**	C	A	S	48	478 T>K
**23017**	T	C	S	1	----
**23031**	T	C	S	1	490 F>S
**23086**	C	T	S	1	----
**23114**	C	T	S	1	----
**23188**	C	T	S	1	----
**23323**	T	C	S	1	----
**23401**	G	T	S	10	----
**23403**	A	G	S	48	614 D>G
**23584**	T	C	S	1	----
**23604**	C	G	S	48	681 P>R
**23609**	C	T	S	1	683 R>W
**23683**	C	T	S	1	----
**23834**	A	G	S	1	758 S>G
**23868**	G	C	S	1	769 G>A
**24095**	G	T	S	1	845 A>S
**24110**	A	C	S	1	850 I>L
**24130**	C	T	S	1	----
**24138**	C	T	S	1	859 T>I
**24175**	T	C	S	1	----
**24368**	G	T	S	1	936 D>Y
**24410**	G	A	S	48	950 D>N
**24707**	C	A	S	1	1049 L>I
**25088**	G	T	S	1	1176 V>F
**25091**	G	T	S	5	1177 V>L
**25135**	G	T	S	1	1191 K>N
**25315**	A	C	S	1	----
**25401**	G	T	ORF3a	1	3 L>F
**25439**	A	C	ORF3a	1	16 K>T
**25445**	G	T	ORF3a	1	18 G>V
**25469**	C	T	ORF3a	48	26 S>L
**25471**	G	T	ORF3a	1	----

**25483**	G	A	ORF3a	1	----
**25513**	C	T	ORF3a	2	41 L>F
**25544**	C	T	ORF3a	1	51 A>V
**25572**	C	T	ORF3a	1	----
**25598**	G	T	ORF3a	1	69 W>L
**25614**	C	T	ORF3a	6	----
**25644**	G	T	ORF3a	1	----
**25658**	C	T	ORF3a	1	89 T>I
**25710**	C	T	ORF3a	1	----
**25806**	A	G	ORF3a	1	----
**25844**	C	T	ORF3a	1	151 T>I
**25906**	G	T	ORF3a	1	172 G>C
**25985**	A	T	ORF3a	2	198 K>I
**26063**	G	T	ORF3a	1	224 G>V
**26109**	G	T	ORF3a	2	239 E>D
**26208**	C	T	ORF3a	1	----
**26370**	C	T	M	1	----
**26421**	C	T	M	1	----
**26526**	G	T	M	5	2 A>S
**26537**	C	T	M	1	----
**26767**	T	C	M	48	82 I>T
**26996**	C	T	M	1	----
**27271**	G	C	ORF6	1	24 V>L
**27346**	T	C	ORF6	1	49 Y>H
**27393**	C	T	ORF7a	1	----
**27516**	G	T	ORF7a	1	----
**27526**	C	T	ORF7a	2	----
**27527**	C	T	ORF7a	24	45 P>L
**27600**	C	T	ORF7a	2	----
**27613**	G	T	ORF7a	4	74 V>F
**27638**	T	C	ORF7a	46	82 V>A
**27739**	C	T	ORF7a	1	116 L>F
**27745**	A	G	ORF7a	1	118 R>G
**27752**	C	T	ORF7a	46	120 T>I
**27754**	G	T	ORF7a	1	121 E>*
**27874**	C	T	ORF7b	46	40 T>I
**27919**	T	C	ORF8	1	9 I>T
**27930**	G	A	ORF8	1	13 V>I

**27945**	C	T	ORF8	2	18 Q>*
**28001**	G	T	ORF8	1	----
**28054**	C	T	ORF8	6	54 S>L
**28075**	G	T	ORF8	3	61 C>F
**28086**	G	T	ORF8	4	65 A>S
**28119**	A	G	ORF8	1	76 I>V
**28299**	A	T	ORF9b	3	6 S>C
**28357**	G	A	ORF9b	4	25 R>K
**28432**	C	T	ORF9b	1	50 S>L
**28461**	A	G	ORF9b	48	60 T>A
**28471**	C	T	ORF9b	1	63 S>F
**28727**	G	T	N	1	152 A>S
**28744**	C	T	N	2	----
**28756**	T	C	N	1	----
**28822**	T	A	N	1	----
**28881**	G	T	N	48	203 R>M
**28887**	C	T	N	1	205 T>I
**28916**	G	T	N	45	215 G>C
**28957**	C	T	N	1	----
**28985**	G	T	N	1	238 G>C
**29009**	G	A	N	1	246 V>I
**29137**	C	T	N	1	----
**29260**	G	T	N	1	----
**29358**	C	T	N	1	----
**29392**	G	T	N	1	373 K>N
**29402**	G	T	N	48	377 D>Y
**29418**	T	C	N	1	382 L>S
**29528**	G	T	N	1	419 A>S
**29540**	G	T	N	1	----
**29557**	G	T	N	1	----
**29690**	G	T	N	1	----
**29710**	T	A	N	1	----
**29726**	T	C	N	1	----
**29742**	G	T	N	48	----

Supplementary Table S2: Overview of detected SNSs (single nucleotide substitutions) in Omicron whole-genome SARS-Cov-2 isolates from Republic of Moldova.

Genomic position in the reference genome	Nucleotide in the reference genome hCoV-19/Wuhan/WIV04/2019|EPI_ISL_402124	Nucleotide in the samples from Republic of Moldova	Genomic region	Number of occurrences of a mutation	Amino acid substitution
**101**	G	A	ORF1a	1	-
**106**	C	T	ORF1a	1	-
**241**	C	T	ORF1a	41	-
**245**	C	A	ORF1a	1	-
**670**	T	G	ORF1a	25	135 S>R
**693**	T	C	ORF1a	1	143 F>S
**945**	G	T	ORF1a	1	227 G>V
**1057**	C	T	ORF1a	1	-
**1150**	C	T	ORF1a	1	-
**1440**	G	A	ORF1a	2	392 G>D
**1471**	C	T	ORF1a	2	-
**1585**	A	C	ORF1a	6	-
**2470**	C	T	ORF1a	2	-
**2583**	C	T	ORF1a	1	773 A>V
**2695**	C	T	ORF1a	1	-
**2721**	C	T	ORF1a	1	819 A>V
**2790**	C	T	ORF1a	25	842 T>I
**2832**	A	G	ORF1a	22	856 K>R
**2902**	C	T	ORF1a	1	-
**3037**	C	T	ORF1a	48	-
**3052**	G	A	ORF1a	1	-
**3140**	C	T	ORF1a	2	959 P>S
**3187**	G	C	ORF1a	1	974 E>D
**3241**	C	T	ORF1a	2	-
**3380**	T	C	ORF1a	1	1039 Y>H
**3409**	A	G	ORF1a	1	-
**3987**	C	T	ORF1a	1	1241 T>I

**4021**	C	T	ORF1a	1	-
**4184**	G	A	ORF1a	25	1307 G>S
**4321**	C	T	ORF1a	21	-
**4331**	C	T	ORF1a	2	-
**4579**	T	A	ORF1a	1	-
**5386**	T	G	ORF1a	22	-
**5672**	C	T	ORF1a	2	1803 P>S
**5730**	C	T	ORF1a	4	1822 T>I
**5746**	C	T	ORF1a	1	-
**5852**	A	G	ORF1a	1	1863 I>V
**5924**	G	A	ORF1a	4	1887 V>I
**6097**	A	G	ORF1a	1	-
**6166**	G	A	ORF1a	1	-
**6191**	A	G	ORF1a	1	1976 T>A
**6402**	C	T	ORF1a	1	2046 P>L
**6723**	C	T	ORF1a	1	2153 T>I
**6996**	T	C	ORF1a	1	2244 I>T
**7037**	G	T	ORF1a	2	2258 G>C
**7324**	T	C	ORF1a	1	-
**7399**	A	G	ORF1a	1	-
**7401**	C	T	ORF1a	1	2379 A>V
**7919**	T	C	ORF1a	1	2552 S>P
**8137**	G	T	ORF1a	1	-
**8393**	G	A	ORF1a	22	2710 A>T
**9344**	C	T	ORF1a	26	3027 L>F
**9424**	A	G	ORF1a	24	-
**9487**	C	T	ORF1a	1	-
**9534**	C	T	ORF1a	25	3090 T>I
**9615**	C	T	ORF1a	1	3117 T>I
**9733**	C	T	ORF1a	1	-
**9866**	C	T	ORF1a	23	3201 L>F
**9890**	G	A	ORF1a	1	3209 A>T
**10021**	A	G	ORF1a	1	-
**10029**	C	T	ORF1a	47	3255 T>I
**10135**	T	C	ORF1a	4	-

**10183**	C	T	ORF1a	1	-
**10198**	C	T	ORF1a	26	-
**10447**	G	A	ORF1a	26	-
**10449**	C	A	ORF1a	48	3395 P>H
**10815**	C	T	ORF1a	1	3517 S>F
**10874**	A	G	ORF1a	1	3537 N>D
**11195**	C	T	ORF1a	1	3644 L>F
**11475**	T	C	ORF1a	1	3737 I>T
**11537**	A	G	ORF1a	22	3758 I>V
**12049**	C	T	ORF1a	2	-
**12676**	C	T	ORF1a	1	-
**12754**	C	T	ORF1a	1	-
**12789**	C	T	ORF1a	2	4175 T>I
**12781**	C	T	ORF1a	1	-
**12880**	C	T	ORF1a	26	-
**12897**	C	T	ORF1a	1	4211 P>L
**13031**	A	T	ORF1a	1	4256 N>Y
**13195**	T	C	ORF1a	22	-
**13521**	T	C	ORF1a	1	-
**13711**	A	G	ORF1b	1	82 K>E
**13994**	C	T	ORF1b	1	176 A>V
**14408**	C	T	ORF1b	48	314 P>L
**14583**	C	T	ORF1b	1	-
**14792**	G	A	ORF1b	1	442 S>N
**14821**	C	T	ORF1b	1	452 P>S
**15063**	A	G	ORF1b	1	-
**15240**	C	T	ORF1b	22	-
**15714**	C	T	ORF1b	26	-
**15924**	C	T	ORF1b	1	-
**16089**	G	A	ORF1b	1	-
**16254**	T	C	ORF1b	1	-
**16289**	C	T	ORF1b	1	941 A>V
**16474**	A	G	ORF1b	1	1003 S>G
**16723**	T	C	ORF1b	1	-
**16740**	A	C	ORF1b	1	1091 E>D

**16866**	T	G	ORF1b	1	-
**16887**	C	T	ORF1b	2	-
**17125**	C	T	ORF1b	1	1220 L>F
**17410**	C	T	ORF1b	26	1315 R>C
**17758**	A	G	ORF1b	1	1431 K>E
**18163**	A	G	ORF1b	48	1566 I>V
**18557**	C	A	ORF1b	1	1697 T>K
**18999**	A	T	ORF1b	1	-
**19602**	C	T	ORF1b	1	-
**19955**	C	T	ORF1b	25	2163 T>I
**20016**	C	T	ORF1b	1	-
**20055**	A	G	ORF1b	26	-
**20364**	A	G	ORF1b	1	-
**20679**	G	A	ORF1b	1	-
**20792**	C	A	ORF1b	1	2442 A>E
**21575**	C	T	S	1	5 L>F
**21595**	C	T	S	1	-
**21618**	C	T	S	26	-
**21682**	C	T	S	1	-
**21762**	C	T	S	18	-
**21846**	C	T	S	18	95 T>I
**21987**	G	A	S	26	142 G>D
**22200**	T	G	S	26	213 V>G
**22207**	T	C	S	1	-
**22237**	A	G	S	1	-
**22578**	G	A	S	48	339 G>D
**22599**	G	A	S	5	346 R>K
**22673**	T	C	S	22	-
**22674**	C	T	S	48	-
**22679**	T	C	S	48	373 S>P
**22686**	C	T	S	48	375 S>F
**22688**	A	G	S	26	376 T>A
**22775**	G	A	S	26	-
**22786**	A	C	S	26	408 R>S
**22792**	C	T	S	18	-

**22813**	G	T	S	48	417 K>N
**22882**	T	G	S	48	440 N>K
**22894**	G	A	S	1	-
**22898**	G	A	S	22	446 G>S
**22992**	G	A	S	48	477 S>N
**22995**	C	A	S	48	478 T>K
**23013**	A	C	S	48	484 E>A
**23040**	A	G	S	48	493 Q>R
**23048**	G	A	S	22	496 G>S
**23055**	A	G	S	48	498 Q>R
**23063**	A	T	S	48	501 N>Y
**23075**	T	C	S	48	505 Y>H
**23202**	C	A	S	22	547 T>K
**23320**	C	T	S	1	-
**23403**	A	G	S	48	614 D>G
**23525**	C	T	S	48	655 H>Y
**23599**	T	G	S	48	-
**23604**	C	A	S	48	681 P>H
**23664**	C	T	S	2	701 A>V
**23673**	C	T	S	1	704 S>L
**23679**	C	T	S	1	706 A>V
**23853**	A	G	S	1	-
**23854**	C	A	S	47	764 N>K
**23948**	G	T	S	47	796 D>Y
**24130**	C	A	S	20	856 N>K
**24385**	A	G	S	1	-
**24424**	A	T	S	47	954 Q>H
**24433**	A	G	S	1	-
**24469**	T	A	S	47	969 N>K
**24503**	C	T	S	19	981 L>F
**24803**	A	G	S	3	1081 I>V
**25000**	C	T	S	48	-
**25157**	G	C	S	1	1199 D>H
**25584**	C	T	ORF3a	48	-
**25596**	A	G	ORF3a	1	-

**25624**	C	T	ORF3a	11	78 Y>H
**25690**	G	C	ORF3a	1	100 G>R
**25708**	C	T	ORF3a	4	106 L>F
**25731**	C	T	ORF3a	2	-
**25751**	T	C	ORF3a	1	120 F>S
**25782**	C	T	ORF3a	1	-
**25855**	G	T	ORF3a	4	155 D>Y
**25906**	G	T	ORF3a	1	172 G>C
**25936**	C	T	ORF3a	1	182 H>Y
**25947**	G	T	ORF3a	1	185 Q>H
**26060**	C	T	ORF3a	26	223 T>I
**26187**	T	C	E	1	-
**26270**	C	T	E	46	9 T>I
**26522**	C	T	M	1	-
**26530**	A	G	M	19	3 D>G
**26577**	C	G	M	46	19 Q>E
**26681**	C	T	M	1	-
**26700**	G	T	M	1	60 V>L
**26709**	G	A	M	46	63 A>T
**26771**	A	G	M	1	-
**26858**	C	T	M	26	-
**26912**	C	T	M	1	-
**27259**	A	C	ORF6	48	-
**27382**	G	C	ORF6	26	61 D>L
**27383**	A	T	ORF6	26	61 D>L
**27384**	T	C	ORF6	28	61 D>L
**27469**	G	A	ORF7a	1	26 G>S
**27807**	C	T	ORF7b	48	-
**28271**	A	T	ORF9b	31	-
**28290**	C	T	ORF9b	1	3 P>S
**28311**	C	T	ORF9b<br>N	32	10 P>S<br>13 P>L
**28612**	A	G	N	1	-
**28826**	C	T	N	2	185 R>C
**28877**	A	T	N	3	-

**28878**	G	C	N	3	-
**28881**	G	A	N	48	203 R>K
**28882**	G	A	N	48	203 R>K
**28883**	G	C	N	48	204 G>R
**29005**	A	T	N	1	244 Q>H
**29095**	C	T	N	1	-
**29179**	G	T	N	1	-
**29253**	C	T	N	1	327 S>L
**29301**	A	G	N	4	343 D>G
**29510**	A	C	N	26	413 S>R
**29568**	T	C	N	1	-
**29632**	C	T	N	3	-
**29700**	A	G	N	1	-
**29744**	G	A	N	1	-
**29760**	T	A	N	3	-

Supplementary Table S3. Detailed information about sequences.

	**Delta (n=48)**			**Omicron (n=48)**		
	**Min**	**Max**	**Average**	**Min**	**Max**	**Average**
**Coverage (%)**	95.2	99.4	98.4	92.9	99.3	96.1
**Ns (missing)**	0	1543	246	11	1422	873
**Frameshift**	0	1	0.06	-	-	-
**Insertions**	-	-	-	0	9	3.4
**Deletions**	13	22	14	18	53	30.3
**AASubstitutions**	26	37	33.4	42	55	48.7
**Aminoacid Deletions**	4	8	4.3	6	16	11.3

Supplementary Table S4. The overall single nucleotide substitution (SNS) transitions (red) and transversions (blue) (SNS = single nucleotide substitution.).

Type of SNS	Delta		Omicron	
	Number (n)	Percentage (%)	Number (n)	Percentage (%)
C > T	125	42.96	92	42.59
G > T	70	24.05	10	3.70
T > C	30	10.31	19	8.80
A > G	26	8.93	32	14.81
G > A	17	5.84	25	11.57
T > A	4	1.37	3	1.39
C > A	4	1.37	8	4.63
A > C	4	1.37	6	2.78
T > G	3	1.03	6	3.70
C > G	3	1.03	1	0.46
A > T	3	1.03	8	2.78
G > C	2	0.69	6	2.78

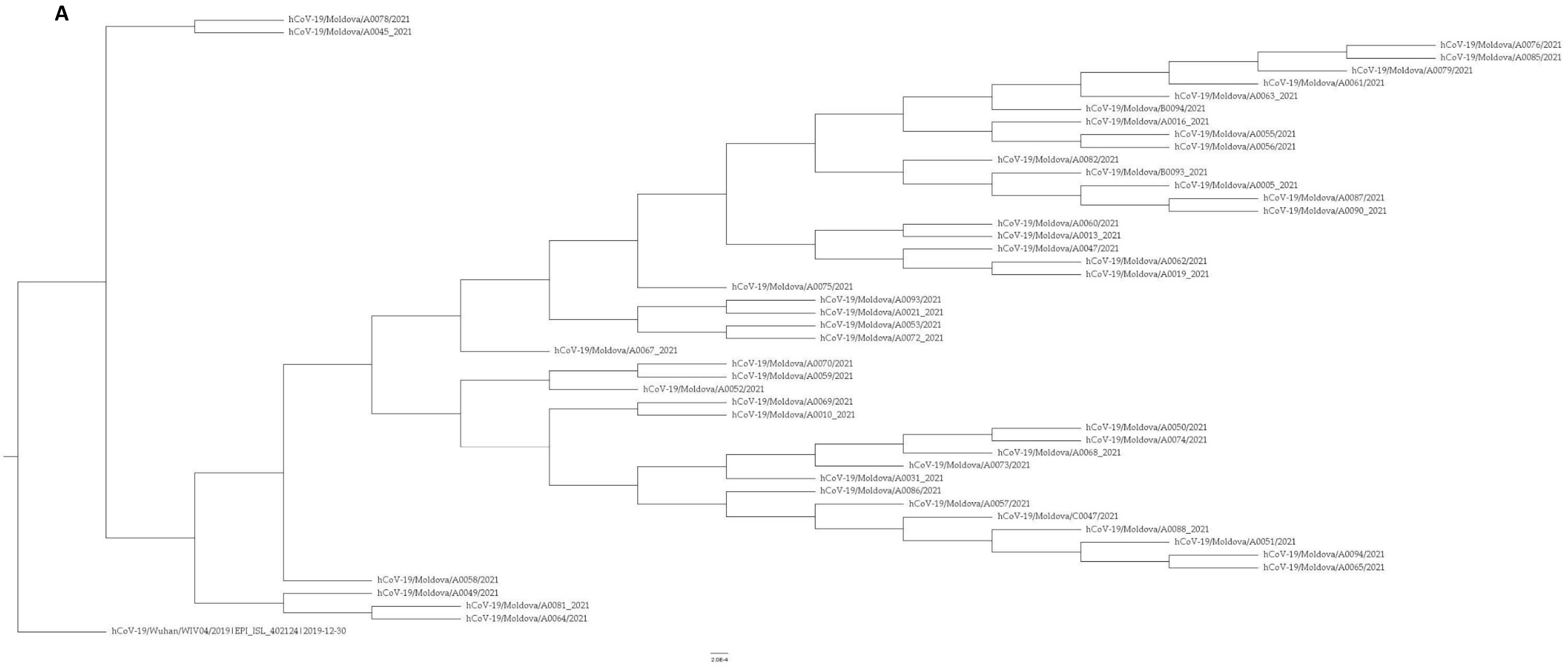

Supplementary **Figure S1.** Phylogenomic analysis of 48 assessed SARS-CoV-2 Delta (A) and Omicron (B) isolates from the Republic of Moldova.

The evolutionary history was inferred by using the Maximum Likelihood method and Tamura-Nei model [13]. The bootstrap consensus tree inferred from 1000 replicates is taken to represent the evolutionary history of the taxa analyzed. Branches corresponding to partitions reproduced in less than 50% bootstrap replicates are collapsed. Initial tree for the heuristic search were obtained by applying the Neighbor-Joining method to a matrix of pairwise distances estimated using the Tamura-Nei model. A discrete Gamma distribution was used to model evolutionary rate differences among sites (5 categories (+G, parameter = 0.1000)). This analysis involved 48 nucleotide sequences. Evolutionary analyses were conducted in MEGA11. Visualization performed in FigTree V.1.4.4.

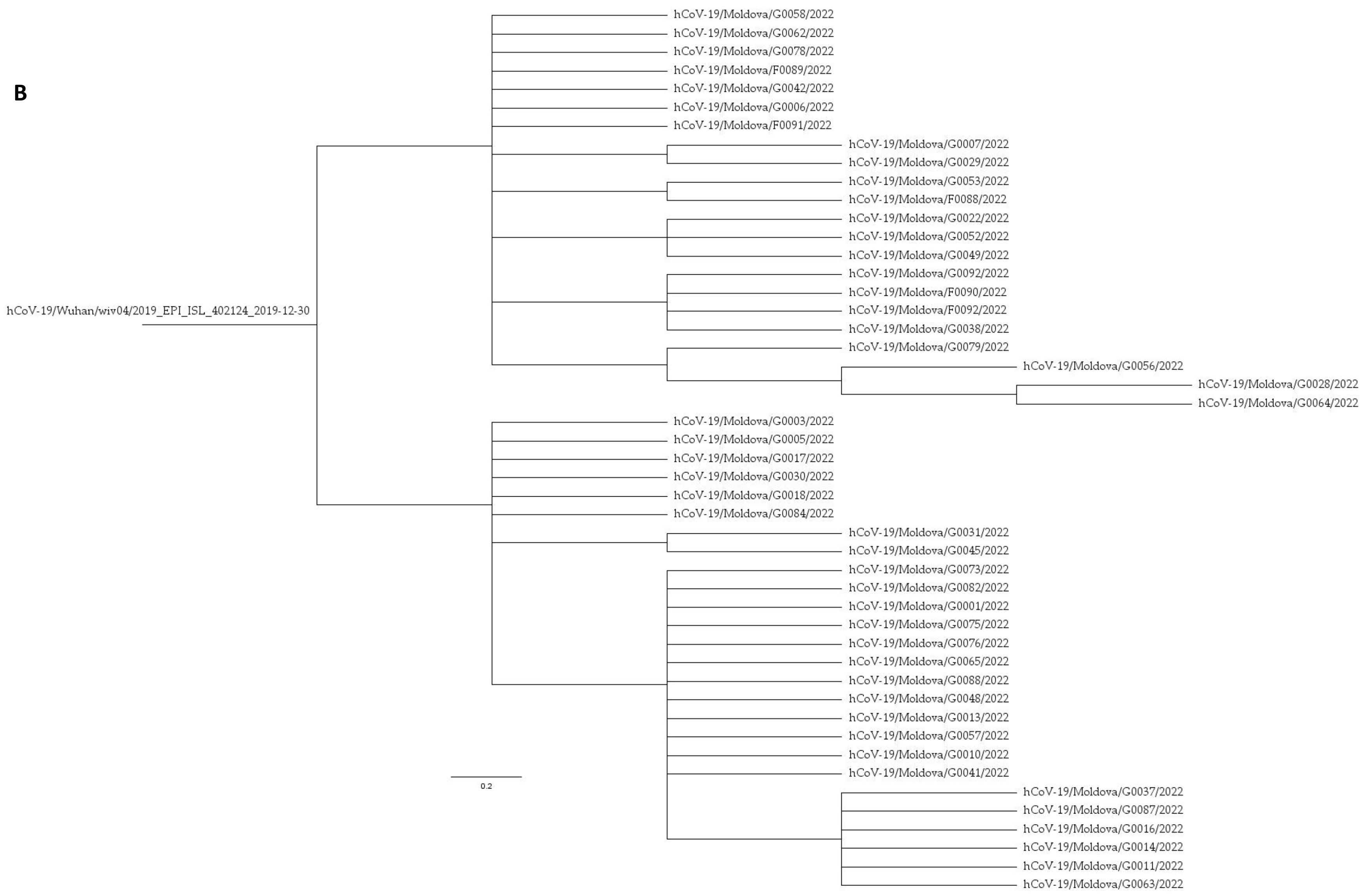
B
hCoV-19/Wuhan/wiv04/2019_EPI_ISL_402124_2019-12-30
hCoV-19/Moldova/G0058/2022
hCoV-19/Moldova/G0062/2022
hCoV-19/Moldova/G0078/2022
hCoV-19/Moldova/F0089/2022
hCoV-19/Moldova/G0042/2022
hCoV-19/Moldova/G0006/2022
hCoV-19/Moldova/F0091/2022
hCoV-19/Moldova/G0007/2022
hCoV-19/Moldova/G0029/2022
hCoV-19/Moldova/G0053/2022
hCoV-19/Moldova/F0088/2022
hCoV-19/Moldova/G0022/2022
hCoV-19/Moldova/G0052/2022
hCoV-19/Moldova/G0049/2022
hCoV-19/Moldova/G0092/2022
hCoV-19/Moldova/F0090/2022
hCoV-19/Moldova/F0092/2022
hCoV-19/Moldova/G0038/2022
hCoV-19/Moldova/G0079/2022
hCoV-19/Moldova/G0056/2022
hCoV-19/Moldova/G0028/2022
hCoV-19/Moldova/G0064/2022
hCoV-19/Moldova/G0003/2022
hCoV-19/Moldova/G0005/2022
hCoV-19/Moldova/G0017/2022
hCoV-19/Moldova/G0030/2022
hCoV-19/Moldova/G0018/2022
hCoV-19/Moldova/G0084/2022
hCoV-19/Moldova/G0031/2022
hCoV-19/Moldova/G0045/2022
hCoV-19/Moldova/G0073/2022
hCoV-19/Moldova/G0082/2022
hCoV-19/Moldova/G0001/2022
hCoV-19/Moldova/G0075/2022
hCoV-19/Moldova/G0076/2022
hCoV-19/Moldova/G0065/2022
hCoV-19/Moldova/G0088/2022
hCoV-19/Moldova/G0048/2022
hCoV-19/Moldova/G0013/2022
hCoV-19/Moldova/G0057/2022
hCoV-19/Moldova/G0010/2022
hCoV-19/Moldova/G0041/2022
hCoV-19/Moldova/G0037/2022
hCoV-19/Moldova/G0087/2022
hCoV-19/Moldova/G0016/2022
hCoV-19/Moldova/G0014/2022
hCoV-19/Moldova/G0011/2022
hCoV-19/Moldova/G0063/2022
0.2

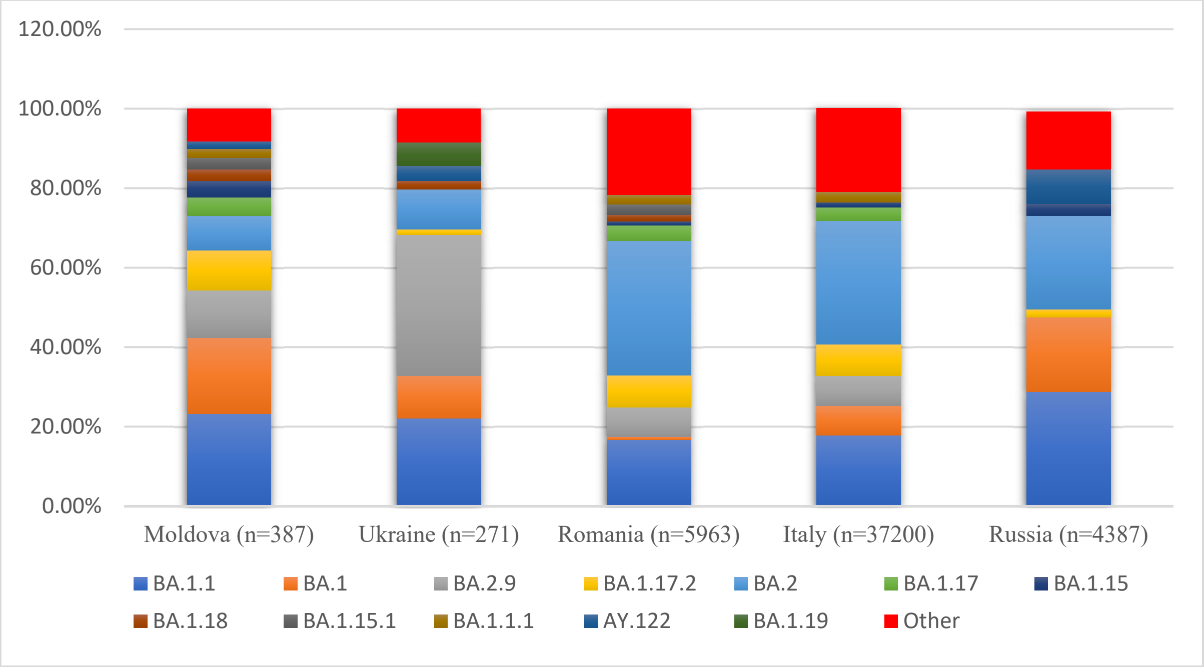

Supplementary Figure S2. Distributions of SARS-CoV-2 lineages in the period January-May 2022.

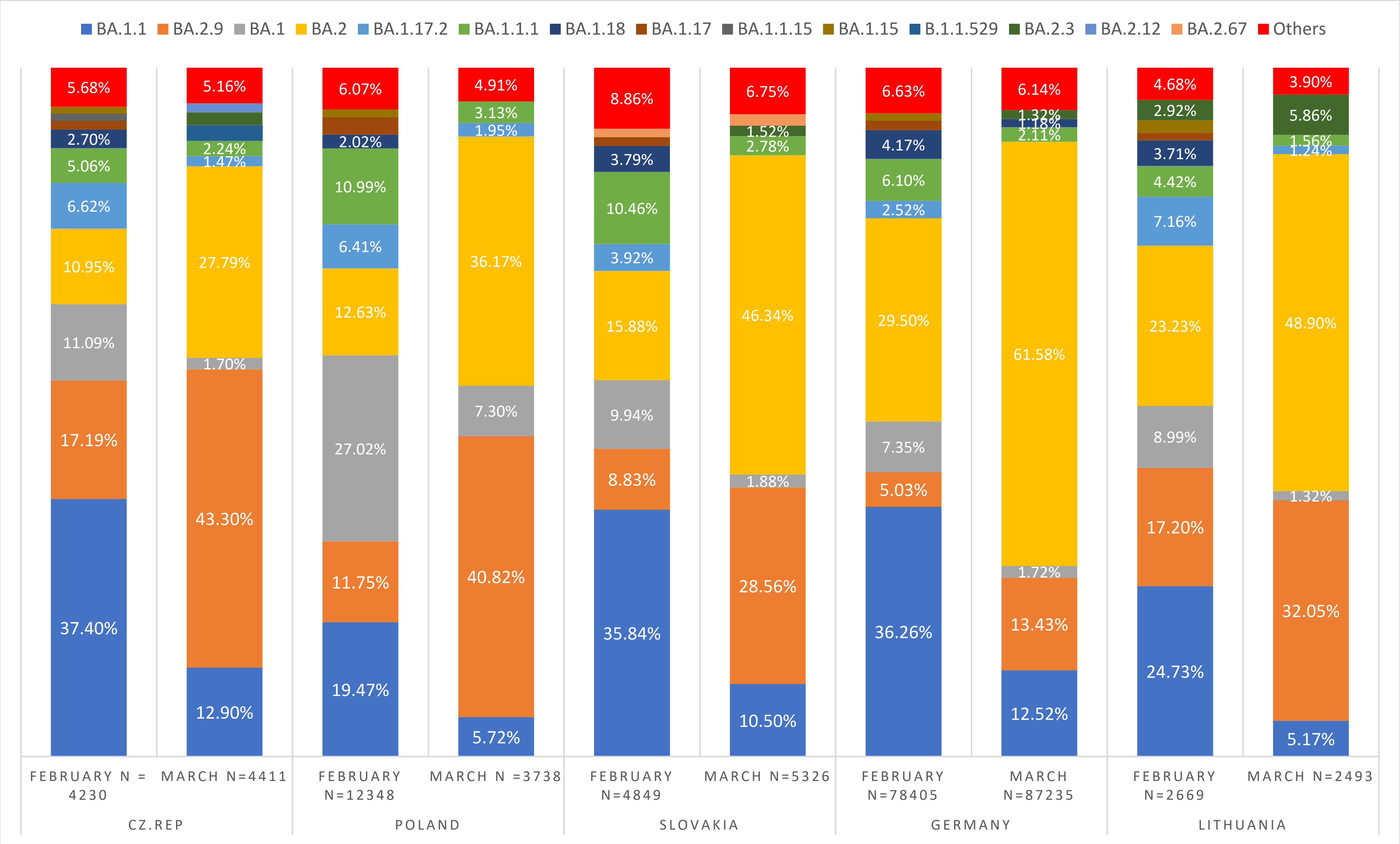

Supplementary Figure S3. Monthly distribution of SARS-CoV-2 lineages between 2 February and March, 2022.
